# Lateralized excitation–inhibition rebalance correlates with motor recovery following hemispheric surgery

**DOI:** 10.1093/braincomms/fcag186

**Published:** 2026-05-23

**Authors:** Yujiao Yang, Kun Lv, Dong Chen, Xiongfei Wang, Chongyang Tang, Jiahui Deng, Xue Yang, Junyuan Chen, Mengyang Wang, Tianfu Li, Jing Wang, Guoming Luan

**Affiliations:** Department of Neurosurgery, Sanbo Brain Hospital, Capital Medical University, Beijing 10093, China; Department of Neurology, Sanbo Brain Hospital, Capital Medical University, Beijing 10093, China; Department of Neurosurgery, Sanbo Brain Hospital, Capital Medical University, Beijing 10093, China; Key Laboratory of Mental Health, Institute of Psychology, Chinese Academy of Sciences, Beijing 100101, China; Department of Neurosurgery, Sanbo Brain Hospital, Capital Medical University, Beijing 10093, China; Department of Neurosurgery, Sanbo Brain Hospital, Capital Medical University, Beijing 10093, China; Department of Neurosurgery, Sanbo Brain Hospital, Capital Medical University, Beijing 10093, China; Department of Neurosurgery, Sanbo Brain Hospital, Capital Medical University, Beijing 10093, China; Department of Neurosurgery, Sanbo Brain Hospital, Capital Medical University, Beijing 10093, China; Department of Neurology, Sanbo Brain Hospital, Capital Medical University, Beijing 10093, China; Department of Neurology, Sanbo Brain Hospital, Capital Medical University, Beijing 10093, China; Department of Neurology, Sanbo Brain Hospital, Capital Medical University, Beijing 10093, China; Department of Neurosurgery, Sanbo Brain Hospital, Capital Medical University, Beijing 10093, China; Beijing Key Laboratory of Epilepsy, Capital Medical University, Beijing 100069, China; Beijing Institute for Brain Disorders, Capital Medical University, Beijing 100069, China; Laboratory for Clinical Medicine, Capital Medical University, Beijing 100069, China

**Keywords:** hemispheric epilepsy surgery, motor recovery, excitation–inhibition balance, aperiodic exponent, neuroplasticity

## Abstract

Hemispheric epilepsy surgery effectively achieves seizure control, yet post-operative motor recovery remains challenging. Whether motor compensation is linked to the excitation–inhibition balance in the unaffected hemisphere (UH) remains unclear. Using scalp EEG, we quantified the aperiodic exponent of the power spectrum as a non-invasive biomarker of cortical excitation–inhibition ratio (where a flatter spectral slope reflects neural excitation). We retrospectively analysed 46 patients who underwent hemispheric surgery and 23 age-matched unilateral non-hemispheric controls. Motor function was measured preoperatively and 3 months post-operatively, with preoperative status classified as preserved or impaired. We assessed preoperative inter-hemispheric asymmetry, post-operative UH modulation and their linear interaction in predicting motor improvement. Jackknife mapping was used to identify the contribution of a specific scalp sensor. Our results show that the hemispheric cohort exhibited a distinct preoperative excitation–inhibition asymmetry (*P* < 0.0001). Post-operatively, the UH exponent interacted significantly with preoperative status to track the change of motor function (*P* < 0.0001; *R*^2^ = 0.53). In patients with preoperative deficits, flatter UH slopes (disinhibition) were associated with functional gains (*P* = 0.0442), while steeper slopes (over-inhibition) correlated with decline (*P* = 0.0001). Conversely, in patients with preserved function, steeper UH slopes (inhibition) were linked to better outcomes (*P* = 0.0023), whereas flatter slopes led to deterioration (*P* = 0.0175). Jackknife mapping localized the primary contribution to the sensor overlying the UH centro-parietal region. These findings suggest that state-dependent modulation of UH excitation–inhibition balance is a factor associated with motor recovery after hemispheric surgery—stabilization is beneficial when baseline function is preserved, while facilitation is beneficial when it is impaired. The aperiodic exponent emerges as a practical biomarker to monitor cortical excitation–inhibition dynamics and to guide individualized, precision neurorehabilitation strategies.

## Teaser

This research explored how excitation–inhibition balance influences motor recovery after hemispheric surgery. Yang and colleagues found that this balance in the unaffected hemisphere significantly impacted motor function. The findings suggest that optimizing it through interventions could guide personalized rehabilitation strategies.

## Graphical Abstract

**Graphical Abstract:**
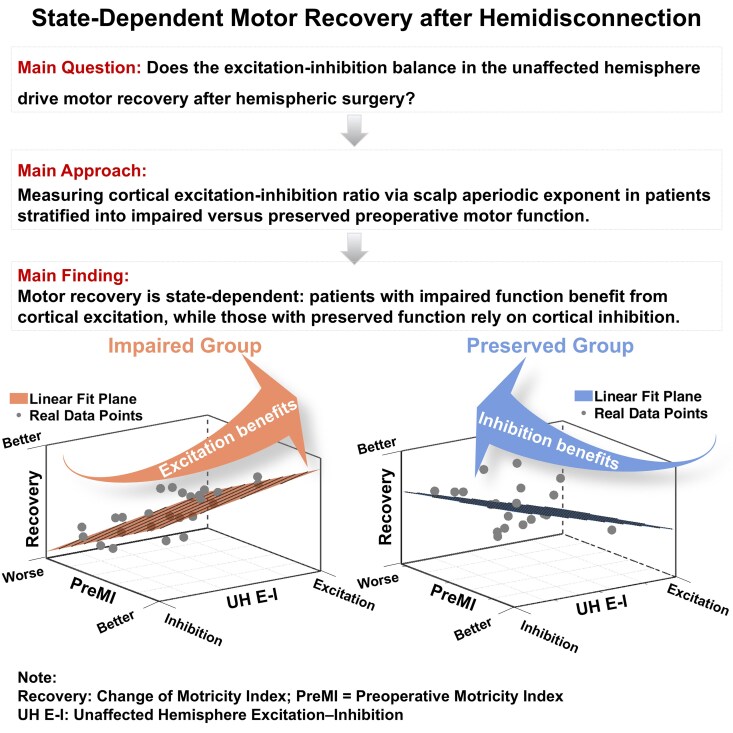

## Introduction

Hemispheric epilepsy surgery is highly effective for controlling drug-resistant seizures,^1,2^ yet the extensive disconnection of corticospinal pathways frequently results in persistent motor deficits.^3,4^ Although many patients show substantial functional recovery—up to 77% of paediatric cases ultimately regain independent ambulation—outcomes are heterogeneous and rarely complete across motor domains.^5^

Recovery therefore relies on adaptive reorganization, principally within the unaffected hemisphere (UH), which must strengthen comparatively weak ipsilateral projections to compensate for disrupted contralateral corticospinal output. Nevertheless, the magnitude of recovery remains difficult to predict. The absence of objective biomarkers to stratify patients and guide personalized rehabilitation constitutes a critical unmet clinical need.

Previous studies have delineated compensatory mechanisms across multiple scales following hemispheric epilepsy surgery. At the molecular and cellular levels, these include corticospinal axonal sprouting and growth,^6,7^ synaptic pruning and local synaptogenesis.^8^ At the mesoscale level, compensatory involves enhanced local projections within motor cortex,^9^ altered neuronal activity patterns^10,11^ and experience-dependent corticospinal plasticity.^11^ At the whole-brain level, investigators have reported interhemispheric rebalancing,^12^ widespread restructuring of structural and functional plasticity,^4,13,14^ changes in functional and network connectivity,^15,16^ and recruitment of alternative motor pathways.^4,17^ Collectively, these findings underscore the remarkable capacity for post-operative neuroplasticity. Yet a critical knowledge gap remains: how intrinsic homeostatic processes in the UH stabilize cortical excitability while enabling adaptive reorganization for motor function.

While classic Hebbian plasticity—activity-dependent synaptic strengthening—has long been recognized as a driver of network adaptation,^18^ Hebbian mechanisms alone can be destabilizing. Without a regulatory counter-mechanism, unchecked synaptic strengthening risks leading to ‘runaway excitation’ and network saturation. This necessitates homeostatic plasticity (HSP), a complementary regulator that dynamically adjusts neuronal excitability and synaptic strengths to maintain a stable set-point of excitation–inhibition (E/I) balance.^19,20^ In the context of brain injury, the ‘bimodal balance-recovery model’ from stroke literature provides a compelling theoretical parallel.^21^ This model posits that motor recovery is jointly determined by structural reserve and interhemispheric balancing. Although hemispherotomy differs anatomically from stroke, the fundamental challenge of managing interhemispheric interactions remains. This motivates a critical inquiry: how does the UH dynamically navigate this plasticity-stability trade-off to maximize motor outcomes in the face of extensive disconnection?

In the context of motor compensation following hemispheric surgery, we propose a novel neuroplasticity mechanism: lateralized HSP (L-HSP). We termed this process ‘L-HSP’ to emphasize that this homeostatic regulation is uniquely driven by the contralesional UH when the affected hemisphere (AH) is functionally isolated. We hypothesize that the UH dynamically retunes its excitability to compensate for the loss of contralateral motor control while preserving network stability. However, the mechanisms of L-HSP and reliable biomarkers that track it remain insufficiently defined, especially how L-HSP navigates the plasticity-stability trade-off across different functional baselines.

To address these challenges, we used non-invasive scalp EEG to examine the aperiodic exponent. The aperiodic exponent, derived from the slope of the 1/*f* component of the EEG power spectrum, has recently emerged as a sensitive indicator of cortical E/I balance.^22^ Physiologically, it reflects the integration of underlying synaptic currents, with recent evidence linking it directly to the ratio of glutamatergic (excitatory) to GABAergic (inhibitory) activity.^23^ A steeper (more negative) exponent typically reflects greater inhibitory tone.^23^ By examining this biomarker, our approach enables direct, *in vivo* assessment of the dynamic regulatory processes underlying post-operative functional recovery.

Given the substantial variability in motor outcomes, we further hypothesize that L-HSP mediates post-operative motor recovery through status-specific mechanisms, consistent with the bimodal balance–recovery framework. Accordingly, this study aims to address two questions: (i) Does the post-operative rebalancing of the UH E/I support motor recovery? (ii) Do the regulatory pathways of L-HSP-mediated motor compensation differ between patients with impaired versus preserved preoperative motor function? By answering these questions, our work aims to clarify the mechanistic role of L-HSP in recovery and to lay the groundwork for more accurate prognostic stratification and individualized neurorehabilitation.

## Materials and methods

### Patient selection

We retrospectively screened all consecutive patients who underwent hemispheric epilepsy surgery at Sanbo Brain Hospital, Capital Medical University, between January 2008 and December 2022. Eligible cases were identified from our institutional epilepsy surgery database. Inclusion criteria were as follows: (1) drug-resistant epilepsy with a unilateral hemispheric aetiology established by comprehensive pre-surgical evaluation and treated with unilateral hemispheric disconnection/resection; (2) seizure freedom at 3-month follow-up; (3) availability of scalp EEG preoperatively and 3 months post-operatively; (4) complete motor function assessments (Motricity Index, MI) at both time points; (5) no structural or electrophysiological evidence of involvement in the UH. Exclusion criteria were (1) prior brain surgery; (2) incomplete or missing follow-up; and (3) poor EEG signal quality. A detailed flowchart of patient recruitment, screening and exclusions is presented in Supplementary Fig. 1. Crucially, to isolate the effects of intrinsic neural plasticity from pharmacological confounds, we confirmed that the anti-seizure medication (ASM) regimen (type and dosage) remained unchanged for all included patients between the preoperative and 3-month post-operative assessments.

To test the specificity of preoperative inter-hemispheric E–I asymmetry, we assembled an age- and sex-matched, unilateral non-hemispheric control cohort from the same time window and databases. Controls met the following criteria: (1) diagnosis of epilepsy with no hemispheric-extent pathology on clinical work-up; (2) no previous epilepsy surgery and no progressive/metabolic disease; and (3) artefact-free baseline scalp EEG.

This study was approved by the Ethics Committee of Sanbo Brain Hospital, Capital Medical University. Written informed consent was obtained from all participants or their legal guardians.

### Operative procedure and follow-up

All surgical decisions and strategies were made through multidisciplinary conferences at the Epilepsy Centre, with the dual aims of achieving complete resection of the epileptogenic zone and maximizing functional preservation. Surgical planning was informed by comprehensive preoperative evaluations, including clinical assessment, scalp EEG, MRI and additional investigations as indicated.

To systematically characterize the cohort heterogeneity, we performed detailed electro-clinical phenotyping. Seizure semiology was classified according to the International League Against Epilepsy (ILAE) 2017 operational classification of seizure types.^24^ Aetiologies were grouped into Acquired, Developmental and Progressive subtypes. Furthermore, EEG features were stratified into three domains: (1) Background Activity (Ipsilateral versus Generalized Slowing); (2) Interictal Epileptiform Discharges (Strictly Unilateral Discharges versus Secondary Bilateral Synchrony); and (3) Ictal Onset Pattern (Regional [≤2 lobes], Multi-lobar/Hemispheric [≥3 lobes], or Non-localizable/Obscured). MRI findings were classified as either regional (involving ≤2 lobes) or multi-regional (>2 lobes).

Two primary surgical procedures were employed: anatomical hemispherectomy and modified hemispherotomy. Anatomical hemispherectomy entailed the complete resection of the AH via a large fronto-temporo-parietal craniotomy extending to the midline. In contrast, modified hemispherotomy was a less invasive, disconnective procedure designed to achieve functional isolation of the epileptogenic hemisphere while preserving maximal cortical tissue. Detailed descriptions of both procedures are available in our previous publication.^25^

All patients were followed up for a minimum of 3 months post-operatively. For this study, only patients achieving seizure freedom at the 3-month follow-up were included in the analysis.

### Motor function assessment

Motor function was quantitatively assessed using the MI,^26^ a standardized and highly reliable clinical scale (range: 0–100), with higher scores reflecting superior motor performance. The MI was evaluated at preoperative baseline and at 3-month post-operatively follow-up by trained clinicians using a consistent protocol.

To analyse state-dependent recovery patterns, patients were stratified based on baseline MI (PreMI) into Preserved (PreMI ≥ 87) and Impaired (PreMI < 87). The cut-off of 87 was derived by subtracting the minimal clinically important difference (MCID) of 13 points from the maximum possible score of 100,^27^ thereby defining the ‘Preserved’ group as those exhibiting no clinically significant motor deficit relative to full function.

**Figure 1:**
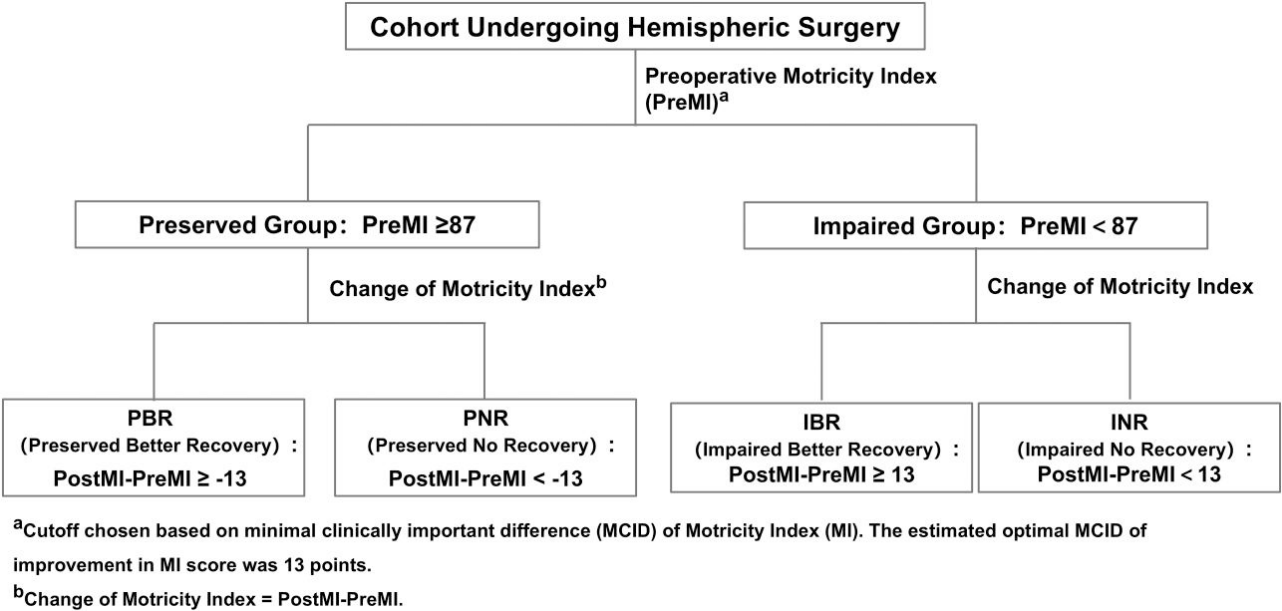
**Patient stratification.** This flowchart details the criteria for patient categorization based on preoperative motor status and post-operative motor recovery. Patients were initially divided into two distinct preoperative status groups based on their baseline Motricity Index (MI) scores: Preserved Group: patients with preoperative MI (PreMI) ≥ 87. Impaired Group: patients with PreMI < 87. Following hemispheric surgery, post-operative motor recovery was assessed using the change of MI (PostMI − PreMI). Better Recovery: defined by a change of MI ≥ 13 points, which represents the Minimal Clinically Important Difference (MCID) for MI improvement. No Recovery: defined by a change of MI < 13 points, indicating no clinically meaningful improvement or a decline in motor function. Combining these two criteria, patients were further stratified into four distinct recovery pattern groups: PBR (Preserved Better Recovery): patients with preserved preoperative motor function who achieved better recovery. PNR (Preserved No Recovery): patients with preserved preoperative motor function who did not achieve better recovery (i.e. experienced decline or no improvement). IBR (Impaired Better Recovery): patients with impaired preoperative motor function who achieved better recovery. INR (Impaired No Recovery): patients with impaired preoperative motor function who did not achieve better recovery.

Post-operative change was defined as ΔMI = PostMI − PreMI. Based on the same MCID threshold of 13 points, patients were further classified into four outcome categories: Preserved–Better Recovery (PBR): Preserved baseline without clinically meaningful decline (ΔMI > −13). Preserved–No Recovery (PNR): Preserved baseline with clinically meaningful decline (ΔMI ≤ −13). Impaired–Better Recovery (IBR): Impaired baseline with clinically meaningful improvement (ΔMI ≥ 13). Impaired–No Recovery (INR): Impaired baseline without clinically meaningful improvement (ΔMI < 13) (Fig. 1).

### EEG recording and pre-processing

Scalp EEG was recorded using a 64-channel Nicolet system, with electrode placement based on the International 10–20 system. Signals were sampled at either 512 or 1024 Hz, with electrode impedance maintained below 5 kΩ. Online referencing was performed to a central channel near Cz. Quantitative analysis focused on the standard 19-channel montage: Fp1/2, F3/4, C3/4, P3/4, O1/2, F7/8, T3/4, T5/6, Fz, Cz and Pz.

To minimize muscle artefacts and state-dependent variability, 5-min artefact-free segments of non-rapid eye movement (NREM) Stage II sleep were selected for analysis. Pre-processing was performed using the EEGLAB toolbox. Raw EEG was re-referenced to the common average of the included electrodes, band-pass filtered from 0.5 to 80 Hz, and down-sampled to 256 Hz. Independent component analysis (ICA) was applied to remove artefacts. Following ICA, all segments underwent rigorous visual inspection, and any epochs with residual artefacts were manually rejected to ensure high signal quality for spectral parameterization. For preoperative recordings, this utilized sensors over both hemispheres. However, for post-operative recordings, channels overlying the AH were excluded prior to this step. Consequently, the post-operative average reference was derived exclusively from the UH sensors, preventing potential artefact contamination from the resection cavity.

### EEG quantitative feature extraction

**Figure 2:**
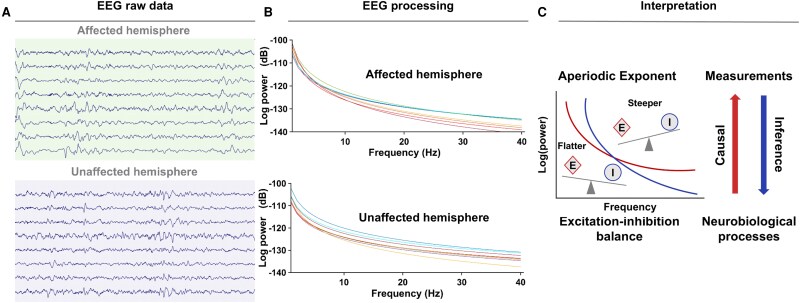
**EEG analysis pipeline.** (**A**) Representative EEG raw data recorded from the Affected Hemisphere (AH) and the Unaffected Hemisphere (UH). (**B**) Power spectral density of EEG signals from the Affected Hemisphere (upper) and Unaffected Hemisphere (lower), plotted as log power (dB) against frequency (Hz). (**C**) Schematic illustration of the theoretical framework. The Aperiodic Exponent, derived from the slope of the 1/f component of the EEG power spectrum, serves as a measurement for inferring the underlying Excitation–Inhibition (E/I) balance within cortical networks. A steeper (more negative) exponent reflects greater inhibitory tone, while a flatter exponent suggests a shift towards greater excitation. Changes in E/I balance as reflected by the Aperiodic Exponent represent the causal neurobiological processes of cortical adaptation.

To characterize cortical excitatory–inhibitory (E/I) dynamics, we extracted the aperiodic exponent of the power spectral density (PSD) (Fig. 2). Based on recent computational and physiological evidence,^22^ the aperiodic exponent is widely utilized as a putative marker tracking the E/I balance of neural circuits.

Aperiodic components were parameterized using the Fitting Oscillations and One-Over-f toolbox.^28^ The fitting range was set from 0.5 to 40 Hz. The ‘knee’ parameter was fixed at zero, modelling the aperiodic component as a linear fit in log–log space. This configuration was supported by the absence of a discernible spectral knee in the 0.5–40 Hz frequency range across the group-averaged power spectra (see Supplementary Fig. S2).

Mathematically, the PSD was modelled as the sum of an aperiodic component L and a periodic oscillatory component:


(1 ) $$PSD=L+\overset{N}{\underset{n=0}{\sum }}\;G_{n}$$

where *N* is the number of peaks, and Gaussians (*G_n_*) represent Gaussian fits to spectral peaks, defined as:


(2 ) $$G_{n}=a\;*\;\exp\;\left(\frac{-{\left(F-c\right)}^{2}}{2w^{2}}\right)$$

where *a* is the amplitude, *c* is the centre frequency, *w* is the bandwidth and *F* is the frequency vector.

The aperiodic signal *L* was modelled as:


(3 ) $$L=b-\log\;(k+F^{\chi })$$

where *b* is broadband offset, *k* is knee parameter and *x* is the exponent. In this study, *F* was set in the range of 0.5–40 Hz, with *k* fixed to 0. In this configuration, the exponent *x* directly corresponds to the negative slope of the PSD = −*a*. A shallower slope (lower exponent value) indicates increased cortical excitability. A representative example of power spectrum parameterization, illustrating the fitting of the aperiodic component to raw power spectral density, is shown in Supplementary Figue 2.

### Statistical analysis

Continuous variables are presented as mean ± SD or median [IQR] depending on normality (assessed via Shapiro–Wilk test) and categorical variables as *n* (%). For baseline clinical characteristics, differences between the Hemispheric and Control groups were examined using Welch’s *t* or Mann–Whitney U-tests for continuous variables, and *χ^2^* or Fisher’s exact tests for categorical variables.

### Hemispheric asymmetry analysis

Crucially, to assess hemispheric asymmetry within each group, we employed paired statistical tests. Within each cohort, the AH versus UH were compared using paired *t*-tests or Wilcoxon signed-rank tests. All tests were two-tailed.

### Linear regression modelling

To investigate whether preoperative motor status modulates post-operative reorganization, we constructed a multiple linear regression model. The primary dependent variable was motor recovery, defined as the change in the MI (ΔMI = PostMI − PreMI). The independent variable of interest was the change of E/I balance within the UH (△Exponent = PostExponent − PreExponent). To prevent overfitting and multi-collinearity issues associated with including multiple electrode-wise predictors, we calculated a single regional aggregate by averaging the exponent across all UH electrodes, generating one composite predictor per patient. This predictor was *z*-standardized prior to modelling.

The model also included relevant covariates: preoperative motor status (binary: Impaired versus Preserved), age at epilepsy onset (*z*-standardized) and aetiology (subgroups: acquired, developmental and progressive). The model was specified as follows:


$$\begin{array}{rl} △MI & =\;\beta 0\;+\;\beta 1\;*\;PreStatus\;+\;\beta 2\;*\;△ExponentUH\\ & \;+\;\beta 3\;*\;AgeOnset\;+\;\beta 4\;*\;Etiology\\ & \;+\;\beta 5\;*\;(PreStatus\;*\;△ExponentUH)\;+\;\varepsilon \end{array}$$

We report *R*^2^, adjusted *R^2^*, the overall *F*-statistic and regression coefficients (*β*) with 95% confidence intervals (CIs). Interaction effects were decomposed using simple slope analysis.

### Sensor-level contribution analysis (Jackknife Method)

To identify which specific scalp regions drove the UH changes, we employed a leave-one-out Jackknife procedure to quantify sensor-level contributions. For channel *k*, the ‘contribution’ was defined as the difference between the grand mean ΔExponent (across all eight UH channels) and the mean recomputed without channel *k*. A positive contribution indicates that the channel pulls the hemispheric mean in the observed direction of change. These contributions were then visualized as channel-wise bar plots and scalp topographies to determine the relative spatial dominance of the electrophysiological shifts.

All statistical analyses were conducted using GraphPad Prism 9 (GraphPad Software, San Diego, CA) and custom MATLAB scripts (MathWorks, Natick, MA). Statistical significance was set at *P* < 0.05. Multiple comparisons were controlled using Benjamini–Hochberg false discovery rate correction, where applicable.

## Results

### Preoperative E/I asymmetry in hemispheric epilepsy

To investigate whether preoperative inter-hemispheric E/I balance is specific to hemispheric pathology, we compared the hemispheric group (HG, *n* = 46) with a matched unilateral non-hemispheric control cohort (*n* = 23). Baseline characteristics were comparable between groups regarding sex, age at seizure onset, age at visit and epilepsy duration (all *P* > 0.05). Detailed clinical characterization, including seizure semiology (focal-to-bilateral versus focal only) and interictal EEG patterns, confirmed that no patients with primary generalized epilepsy were included (Table 1 and Supplementary Table 1).

**Table 1:** Clinical characteristics of patients across different groups

	Hemispheric Group (*N* = 46)	Control Group (*N* = 23)	*P-*value
Gender (male/female)	23/23(50.0%, M)	12/11(52.2%, M)	0.87
Age at seizure onset (years)	2.9 ± 5.1	3.9 ± 2.2	0.26
Age at visit (years)	5.9 ± 6.7	5.7 ± 2.0	0.85
Epilepsy duration (years）	3.0 ± 3.8	1.9 ± 1.6	0.10
Seizure type			0.86
Focal	29(63.0%)	14(60.9%)	
General	17(37.0%)	9(39.1%)	
Interictal EEG distribution			0.86
Regional	31(67.4%)	15(65.2%)	
Multiregional	15(32.6%)	8(34.8%)	
Ictal EEG distribution			0.17
Regional	24(52.2%)	16(69.6%)	
Multiregional	22(47.8%)	7(30.4%)	
MRI lesion burden			<0.0001*
Negative	0(0.0%)	9(39.1%)	
MRI positive	46(100%)	14(60.9%)	
Regional	11(23.9%)	9(39.1%)	
Multi-regional	35(76.1%)	5(21.7%)	

[*]Data are presented as *n* (%) for categorical variables or mean ± standard deviation (SD) for continuous variables.

**Figure 3:**
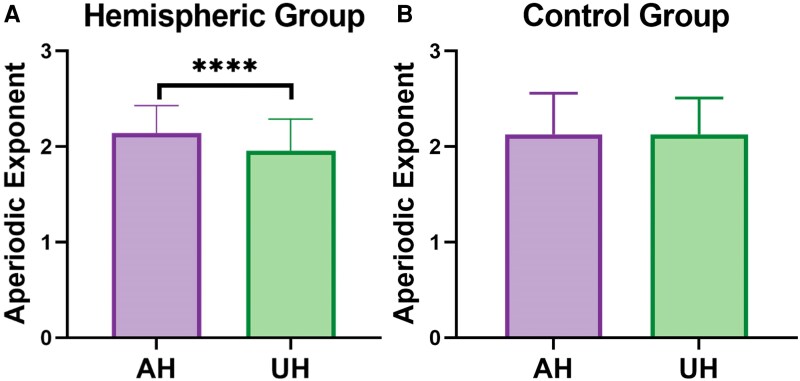
**Preoperative excitation–inhibition (E/I) asymmetry in hemispheric epilepsy.** (**A**) Preoperative aperiodic exponents in Hemispheric Group (*n* = 46). The aperiodic exponent for the affected hemisphere (AH) and unaffected hemisphere (UH) has a significant lateralized asymmetry (mean ± SD: 2.14 ± 0.29 versus 1.96 ± 0.33; paired *t*-test, *t*_(45)_ = 5.25, *P* < 0.0001). (**B**) Preoperative aperiodic exponents in Control Group (*n* = 16). No significant asymmetry is observed, with similar values (mean ± SD: 2.13 ± 0.43 versus 2.13 ± 0.39; paired *t*-test, *t*  _(22)_ = 0.10, *P* = 0.9785). *P-*values: *P < 0.05; **P < 0.01; ***P < 0.001; ****P < 0.0001.

Crucially, only the HG displayed a lateralized preoperative aperiodic exponent: the AH significantly exceeded the UH (mean ± SD: 2.14 ± 0.29 versus 1.96 ± 0.33; paired *t*-test, *t*_(45)_ = 5.25, *P* < 0.0001; Fig. 3A). In contrast, the control group exhibited no hemispheric asymmetry (mean ± SD: 2.13 ± 0.43 versus 2.13 ± 0.39; paired *t*-test, *t*_(22)_ = 0.10, *P* = 0.9785; Fig. 3B).

To address the potential confounding effect of focal lesions within the control cohort, we further stratified the controls into MRI-negative (*n* = 9) and MRI-positive (*n* = 14) subgroups. This analysis revealed that inter-hemispheric symmetry was preserved regardless of lesion status: neither the MRI-negative subgroup (mean ± SD: 2.10 ± 0.50 versus 2.09 ± 0.43; paired *t*-test, *t*_(8)_ = 0.20, *P* = 0.8458; Supplementary Fig. 3Middle) nor the MRI-negative subgroup (mean ± SD: 2.03 ± 0.25 versus 2.03 ± 0.25; paired *t*-test, *t*_(13)_ = 0.48, *P* = 0.6360; Supplementary Fig. 3, Right).

These results demonstrate that preoperative inter-hemispheric E/I imbalance is a distinctive hallmark of hemispheric epilepsy, which is absent in unilateral non-hemispheric epilepsy.

### Preoperative status modulates post-operative reorganization

**Figure 4:**
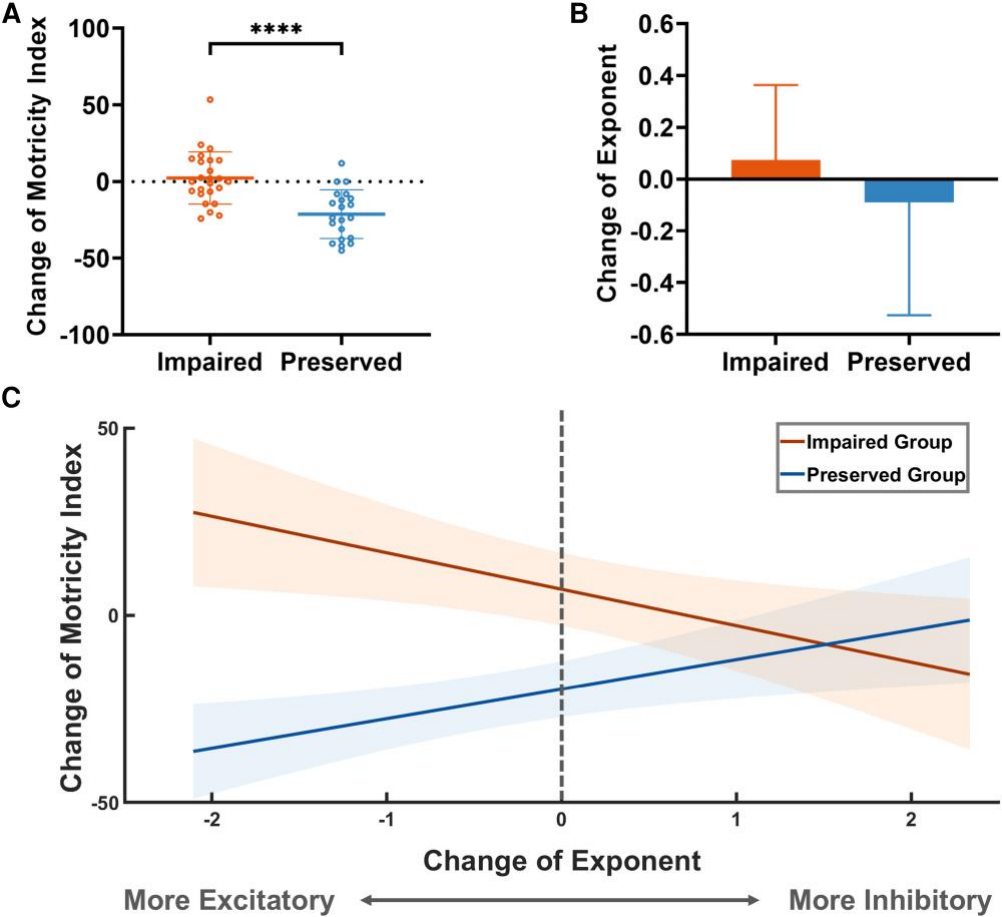
**Interaction between preoperative status and UH exponent.** (**A**) Change of Motricity Index (ΔMI) between the Impaired (*n* = 25) and Preserved (*n* = 21) groups. Each data point represents an individual patient. The Impaired group demonstrated a significant improvement compared to the Preserved group (mean ± SD: 2.40 ± 17.03 versus −21.21 ± 15.94; unpaired *t*-test, *t*(44) = 4.82, *P* < 0.0001). (**B**) Change of unaffected hemisphere (UH) aperiodic exponent (ΔExponent) between the Impaired and Preserved groups. No significant difference was observed (mean ± SD: 0.07 ± 0.29 versus −0.09 ± 0.44; unpaired *t*-test, *t*(44) = 1.53, *P* = 0.1335). (**C**) This interaction plot illustrates the predicted change of Motricity Index as a function of the *z*-standardized change of exponent from the linear regression model. Lines represent the simple slopes for the Impaired group (orange) and Preserved group (blue), with shaded areas indicating 95% confidence intervals. The vertical dashed line at 0 delineates the transition from more excitation (ΔExponent < 0, left) to more inhibition (ΔExponent > 0, right). The significant interaction (*t*(39) = 3.61, *P* = 0.0006) underscores divergent pathways: the Preserved group benefits from inhibitory shifts while the Impaired group shows improved recovery with excitatory shifts. *P-*values: *P < 0.05; **P < 0.01; ***P < 0.001; ****P < 0.0001.

We first contrasted post-operative change between the Impaired and Preserved cohorts. For the change of MI (△MI), the Impaired group (*n* = 25) showed markedly greater improvement than the Preserved group (*n* = 21) (mean ± SD: 2.40 ± 17.03 versus −21.21 ± 15.94; unpaired *t*-test, t(44) = 4.82, *P* < 0.0001) (Fig. 4A). For the change of aperiodic exponent (△Exponent), no significant difference was observed between groups (mean ± SD: 0.07 ± 0.29 versus −0.09 ± 0.44; unpaired *t*-test, *t*(44) = 1.53, *P* = 0.1335) (Fig. 4B).

To rigorously test the state-dependent hypothesis while ruling out potential confounds from etiologic heterogeneity, we fit a multivariable linear regression model predicting ΔMI. The model included the change in UH exponent (ΔExponent), preoperative status, and their interaction, while strictly controlling for age at onset and aetiology (stratified into three distinct subgroups: acquired, progressive and developmental). The model explained a substantial proportion of variance in △MI (*R*^2^ = 0.54, adjusted *R*^2^ = 0.47; *F*_(6,39)_ = 7.68, *P* = 1.74 × 10^−5^). Crucially, the interaction between preoperative status and ΔExponent remained highly significant even after adjusting for specific etiologic subgroups (*β* = 17.63, *SE* = 4.80, *t* = 3.61, *P* = 0.0009), indicating that the behavioural relevance of UH rebalancing depends on baseline status.

Covariates showed no independent effects: age at onset (*β* = −3.58, *P* = 0.1532) and aetiology (progressive versus acquired, *β* = −5.63, *P* = 0.31; developmental versus acquired, *β* = 0.12, *P* = 0.9894). A main effect of status remained significant (*β* = −24.06, *P* = 1.88 × 10^−5^), consistent with different recovery potential at baseline.

Within-status simple-slope estimates confirmed the reversal effect: in the Preserved group, increased inhibition (higher ΔExponent) predicted better outcomes (*β* = +7.86, *P* = 0.0085; 95% CI: 2.30 to 13.41), whereas in the Impaired group, increased disinhibition (lower ΔExponent) predicted greater improvement (*β* = −9.45, *P* = 0.0190; 95% CI: −17.02 to −1.88).

Together, these findings support a state-dependent framework for reorganization: favourable outcomes in Preserved patients were associated with a steepening of the aperiodic slope (suggestive of stabilized excitability), whereas improvement in Impaired patients tracked with spectral flattening (consistent with increased excitability).

### Post-operative modulation in the preserved group

To identify the contribution of specific electrodes to the spectral shift, we stratified the Preserved Group by outcome. Given the high baseline function in this cohort, improvement was constrained by a ceiling effect; thus, ‘Better Recovery’ in this context reflects the successful maintenance of function against surgical perturbation.

**Figure 5:**
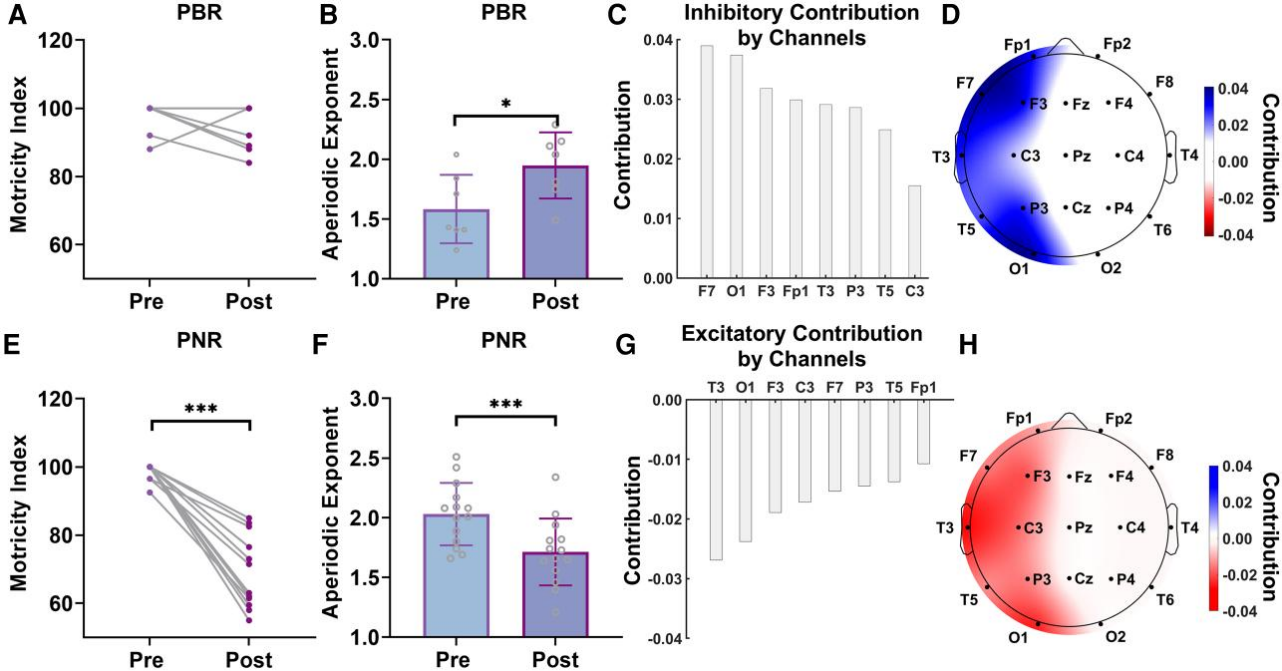
**Post-operative E/I rebalance and motor outcomes in the preserved group.** (**A**) Change of Motricity Index from pre- to post-operative assessment in the Preserved Better Recovery (PBR) group (*n* = 7), presented as individual data points (each representing an individual patient) with connecting lines, indicating maintenance or improvement in motor performance (Wilcoxon matched-pairs signed rank test, *W* = −6.00, *P* = 0.5625). (**B**) Post-operative change in unaffected hemisphere (UH) aperiodic exponent in the PBR group, showing a significant increase, reflecting a shift in the excitation-inhibition (E/I) balance towards inhibition (mean ± SD: 1.58 ± 0.29 versus 1.95 ± 0.28; paired *t*-test, *t*(6) = 2.54, *P* = 0.0442). Each scatter point represents an individual patient. (**C**) Channel-Wise contributions to UH E/I shift towards inhibition in the PBR group (*n* = 7 patients). The bar graph illustrates the contributions of EEG channels to the post-operative change in UH aperiodic exponent. The inhibition was primarily driven by contributions from the F7 and O1, while the C3 electrode exhibited the least inhibitory influence. (**D**) Topographic representation of channel contributions in the PBR group (*n* = 7 patients). This circular head plot illustrates the spatial distribution of channel contributions. The UH was mapped to the left side of the scalp. Blue shades represent the degree of inhibitory contribution, with deeper blue indicating stronger inhibition, reflecting the beneficial effect of inhibition on motor recovery in the PBR group. Key electrodes (F7, O1) are marked, highlighting that while the fronto-occipital network predominantly drives this inhibitory shift, the C3 electrode exhibits the least inhibitory influence. (**E**) The change in Motricity Index from pre- to post-operative assessment in the Preserved No Recovery (PNR) group (*n* = 14), depicted as individual data points (each representing an individual patient) with connecting lines, illustrating a significant reduction in motor performance (Wilcoxon matched-pairs signed rank test, *W* = −105.0, *P* = 0.0001). (**F**) The post-operative change of UH aperiodic exponent in the PNR group. A significant decrease towards disinhibition is observed (mean ± SD: 2.03 ± 0.26 versus 1.71 ± 0.28; paired *t*-test, *t*(13) = 4.97, *P* = 0.0003). Each scatter point represents an individual patient. (**G**) The bar graph highlights the contributions of individual EEG channels to the post-operative decrease in UH aperiodic exponent in the PNR group (*n* = 14 patients), indicating a shift towards excitation. Contributions are shown for key electrodes (T3, O1, F3, C3), with negative values reflecting a driving effect towards increased excitation, as determined by jackknife resampling. (**H**) The circular head plot depicts the spatial distribution of channel contributions (*n* = 14 patients), with the UH mapped to the left side of the scalp. Red shading indicates the degree of excitatory contribution, with deeper red signifying stronger excitation, reflecting the detrimental impact of disinhibition on motor recovery. Key electrodes (T3, O1, F3, C3) are marked, emphasizing their predominant role in driving this excitatory shift. *P* values: *P < 0.05; **P < 0.01; ***P < 0.001; ****P < 0.0001.

Patients who maintained optimal function (Preserved Better Recovery, PBR, *n* = 7) demonstrated a significant increase in the UH aperiodic exponent (mean ± SD: 1.58 ± 0.29 preoperatively versus 1.95 ± 0.28 post-operatively; Paired *t*-test, *t*(6) = 2.54, *P* = 0.0442) (Fig. 5B). Their motor performance was effectively maintained (MI: 97.14 ± 5.01 preoperatively versus 93.29 ± 6.70; Wilcoxon matched-pairs signed rank test, *W* = −6.00, *P* = 0.5625) (Fig. 5A). Crucially, Jackknife-based topographical analysis revealed that the contribution to this spectral slope steepening was weighted primarily towards F7 and O1 electrodes, with the least contribution from C3 (Fig. 5C and D). This spatial dissociation suggests that while broad scalp regions shifted towards inhibition (stabilization), excitability over the motor cortex was relatively preserved, thereby supporting continued motor execution.

By contrast, patients suffered functional decline (Preserved No Recovery, PNR, *n* = 14) exhibited a significant decrease in the UH aperiodic exponent (mean ± SD: 2.03 ± 0.26 preoperatively versus 1.71 ± 0.28 post-operatively; paired *t*-test, *t*(13) = 4.97, *P* = 0.0003) (Fig. 5F). This spectral flattening (suggesting disinhibition) was accompanied by a marked MI decline (mean ± SD: 98.96 ± 2.25 preoperatively versus 69.07 ± 10.46; Wilcoxon matched-pairs signed rank test, *W* = −105.0, *P* = 0.0001) (Fig. 5E). Jackknife analysis implicated that this excitatory shift was widespread, with significant contributions from T3, O1, F3 and notably C3 (Fig. 5G and H). Unlike the PBR group, the involvement of sensors in this broad disinhibition points to a loss of focal control, where excessive excitability disrupts the stability required to maintain motor output.

### Post-operative modulation in the impaired group

**Figure 6:**
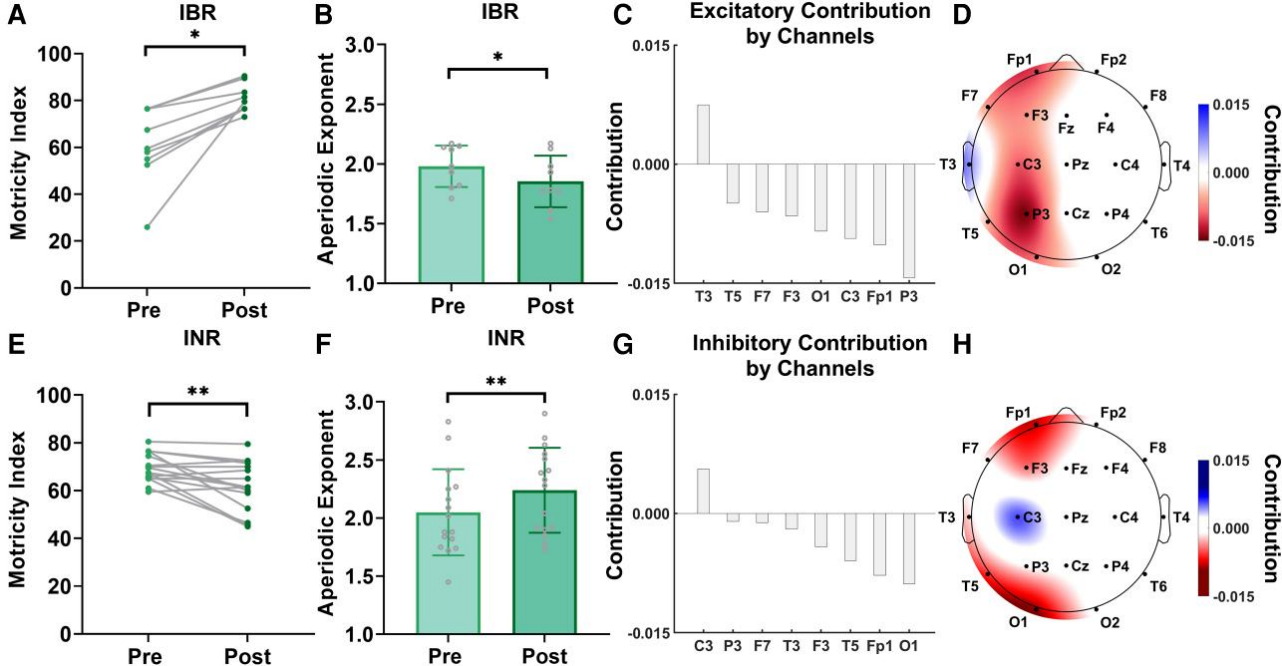
**Post-operative E/I rebalance and motor outcomes in the impaired group.** (**A**) Motor Performance Improvement in the Impaired Better Recovery (IBR) group. This panel displays the change in Motricity Index from pre- to post-operative assessment in the IBR group (*n* = 9), presented as individual data points (each representing an individual patient) with connecting lines, showing a significant improvement in motor performance (mean ± SD: 60.89 ± 16.23 versus 80.18 ± 6.08; paired *t*-test, t (8) = 4.41, *P* = 0.0023). (**B**) The postoperative change in unaffected hemisphere (UH) aperiodic exponent in the IBR group. Each scatter point represents an individual patient. A significant decrease towards enhanced excitation is observed, reflecting a shift in the excitation-inhibition (E/I) balance towards excitation (mean ± SD: 1.98 ± 0.17 versus 1.85 ± 0.27; paired *t*-test, t (8) = 2.98, *P* = 0.0175). (**C**) Channel-Wise Contributions to UH E/I shift towards excitation in the IBR Group (*n* = 9 patients). The bar graph highlights the contributions of EEG channels to the postoperative decrease in UH aperiodic exponent in the IBR group, indicating a shift towards excitation. Contributions are shown for key electrodes (P3, Fp1, C3), with negative values reflecting a driving effect towards increased excitation, as determined by jackknife resampling. (**D**) Topographic Representation of Channel Contributions (*n* = 9 patients). This circular head plot depicts the spatial distribution of channel contributions in the IBR group, with the UH mapped to the left side of the scalp. Red shading indicates the degree of excitatory contribution, with deeper red signifying stronger excitation, reflecting the beneficial impact of this shift on motor recovery. Key electrodes (P3, Fp1, C3) are marked, emphasizing their predominant role in driving excitatory rebalance. (**E**) Motor Performance Decline in the Impaired No Recovery (INR) group. This panel presents the change in Motricity Index from pre- to postoperative assessment in the INR group (*n* = 16), depicted as individual data points (each representing an individual patient) with connecting lines, illustrating a significant decline in motor performance (mean ± SD: 69.47 ± 6.02 versus 62.03 ± 10.45; paired *t*-test, t (15) = 3.34, *P* = 0.0045). (**F**) Postoperative E/I Inhibition in the INR Subgroup. This boxplot illustrates the post-operative change in UH aperiodic exponent. A significant increase towards inhibition is observed (mean ± SD: 2.05 ± 0.37 versus 2.24 ± 0.37; paired *t*-test, t (15) = 2.57, *P* = 0.0215). Each scatter point represents an individual patient. (**G**) Channel-Wise Contributions to UH E/I Inhibition in the INR Group (*n* = 16 patients). This bar graph highlights the contributions of EEG channels to the post-operative increase in UH aperiodic exponent, indicating a shift towards inhibition. Contributions are shown for key electrodes (C3), with positive values reflecting a driving effect towards increased inhibition, as determined by jackknife resampling. (**H**) Topographic Representation of Channel Contributions in the INR Group (*n* = 16 patients). This circular head plot depicts the spatial distribution of channel contributions to the post-operative increase in UH aperiodic exponent, with the UH mapped to the left side of the scalp. Blue shading indicates the degree of inhibitory contribution, with deeper blue signifying stronger inhibition, reflecting the detrimental impact of this shift on motor recovery. The C3 electrode is marked, emphasizing its predominant role in driving inhibitory rebalance. *P* values: *P < 0.05; **P < 0.01; ***P < 0.001; ****P < 0.0001.

In the Impaired Group, patients who achieved better recovery (Impaired Better Recovery, IBR, *n* = 9) exhibited a significant decrease in the UH aperiodic exponent (mean ± SD: 1.98 ± 0.17 preoperatively versus 1.85 ± 0.27 post-operatively; Paired *t*-test, *t*(8) = 2.98, *P* = 0.0175) (Fig. 6B). This spectral flattening coincided with substantial improvement in motor performance (MI mean ± SD: 60.89 ± 16.23 preoperatively versus 80.18 ± 6.08; Paired *t*-test, *t*(8) = 4.41, *P* = 0.0023) (Fig. 6A). Jackknife analysis highlighted that the contribution to this excitatory shift was weight towards P3, Fp1 and notably C3 electrodes (Fig. 6C and D). This involvement of the central sensor suggests that, unlike the Preserved group, where stability was key, recovery in the Impaired group benefited from a disinhibitory shift over the motor area to facilitate recruitment.

Conversely, patients who failed to recover (Impaired No Recovery, INR, *n* = 16) exhibited a significant post-operative increase in the UH aperiodic exponent (mean ± SD: 2.05 ± 0.37 preoperatively versus 2.24 ± 0.37 post-operatively; Paired *t*-test, *t*(15) = 2.57, *P* = 0.0215) (Fig. 6F). This shift towards inhibition with accompanied by a significant decline in MI (mean ± SD: 69.47 ± 6.02 preoperatively versus 62.03 ± 10.45 post-operatively; Paired *t*-test, *t*(15) = 3.34, *P* = 0.0045) (Fig. 6E). Jackknife analysis indicated that C3 electrode contributed positively to this steepening (Fig. 6G and H), suggesting that inhibition over the motor cortex—effectively ‘silencing’ the sensorimotor output—may underlie the functional deterioration observed in these patients.

## Discussion

**Figure 7:**
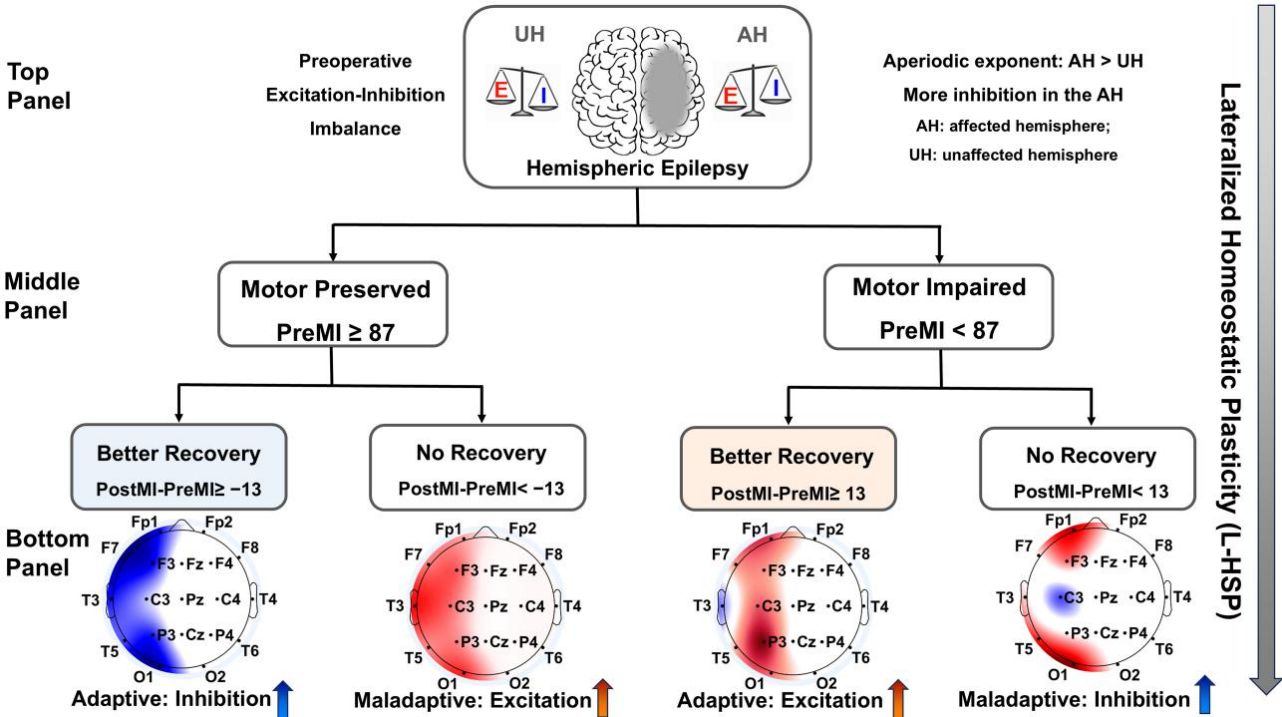
**Summary of lateralized homeostatic plasticity (L-HSP) pathways underlying motor recovery.** Top Panel: Preoperative Inter-hemispheric E/I Imbalance. In patients with hemispheric epilepsy, the Affected Hemisphere (AH) typically exhibits a state of more inhibition compared to the Unaffected Hemisphere (UH) as reflected by the Aperiodic Exponent (AH > UH). Middle Panel: Preoperative Motor Status Stratification. Patients are stratified into two Preoperative Motor Status Groups based on their baseline Motricity Index (PreMI) scores: Motor Preserved Group: Patients with PreMI ≥ 87. Motor Impaired Group: Patients with PreMI < 87. Bottom Panel: Four Distinct Post-operative E/I Rebalancing Patterns. Following hemispheric surgery, the UH undergoes various E/I rebalancing, leading to four distinct patterns that correlate with motor recovery outcomes, as measured by the post-operative Motricity Index (PostMI). In the Preserved Group: patients who achieve better recovery exhibit an adaptive increase in inhibition within the UH. The topographical map illustrates the key regions contributing to this increased inhibitory tone. This represents an adaptive rebalancing for this status group. Patients who show no recovery demonstrate a maladaptive increase in excitation within the UH. The topographical map highlights the regions where this shift towards excitation is most prominent, leading to poor outcomes. In the Impaired Group: patients who achieve better recovery exhibit an adaptive increase in excitation within the UH. The topographical map points to the regions driving this excitatory shift, crucial for recovery in this status group. Patients who show no recovery demonstrate a maladaptive increase in inhibition within the UH. The topographical map indicates regions where excessive inhibition is detrimental to recovery.

Recent work highlights a central role of the UH in motor reorganization following hemispheric surgery^13,14^; however, the underlying neuroplastic mechanisms remain to be fully elucidated. Our findings advance this understanding by linking EEG PSD spectral changes to functional recovery. First, a preoperative inter-hemispheric E/I asymmetry appears characteristic of hemispheric epilepsy: only the hemispheric cohort showed a lateralized aperiodic exponent (AH > UH), whereas a matched unilateral non-hemispheric control cohort exhibited no such asymmetry. Second, post-operative motor recovery is state-dependent: a moderation model demonstrated a robust interaction between preoperative status and changes in the UH aperiodic exponent. Specifically, flattening of the slope (suggesting increased E/I ratio) predicted improvement in impaired patients, while steepening of the slope (suggesting stabilization) predicted better outcomes in preserved patients. Third, stratified analyses revealed four reproducible remodelling profiles (PBR, PNR, IBR and INR) that map onto a contralesional centro-parietal sensor locations. Together, these results outline a potential framework—from preoperative hemispheric E/I imbalance, through state-dependent UH remodelling, to motor outcome—which may inform future individualized post-operative rehabilitation (Fig. 7).

Neuroplasticity is the capacity of the nervous system to adapt and reorganize in response to stimuli, injury or intervention, which is crucial for recovery.^13,29,30^ Motor reorganization after hemispheric surgery is hypothesized to be governed not only by Hebbian learning but also by HSP, which continuously retunes cortical excitability towards a set-point.^31^ Within this control scheme, the UH operates as the principal compensator^15^ and likely adjusts its excitability to support network rebalance. Our data indicate that this adjustment is state-dependent: functional maintenance (limiting the inevitable surgical decline) aligns with spectral stabilization in patients with preserved baseline function, whereas effective recovery aligns with spectral facilitation in those with impaired function. We term this mechanism L-HSP and propose it as a key correlate of post-operative motor recovery, serving distinct neuroprotective or restorative roles depending on the preoperative baseline.

Our observation of distinct patterns in homeostatic regulation aligns with the established literature on post-stroke recovery,^21,31,32^ particularly regarding the critical role of E/I rebalancing in functional outcomes. Like the bimodal balance–recovery model proposed for stroke neurorehabilitation^21^: when structural baseline function is relatively preserved, recovery follows a balance regime in which stabilizing interhemispheric interactions—typically by increasing inhibition of the contralesional hemisphere—is advantageous; when reserve is poor, recovery follows a recovery regime, whereby facilitation of the contralesional hemisphere unlocks compensatory resources. However, a critical distinction in hemispheric lesion is that the AH is largely disconnected or resected; thus, the primary locus for compensation inevitably shifts entirely to the contralesional UH. Applying this framework to our data: in our Impaired cohort, the IBR subgroup exhibited beneficial motor recovery alongside a flattening of the spectral slope (disinhibition) in the UH over the fronto-parietal territory. Conversely, INR showed maladaptive decline with steepening slopes (over-inhibition) centred on motor-related region (C3), consistent with a failure to release compensatory drive. In the Preserved cohort, the pattern inverted: PBR maintained motor performance while the UH became more inhibitory; PNR, in turn, lost inhibitory tone in the same hub and deteriorated. These outcomes tied to opposite E/I directions operationalize the bimodal model within the UH: state-appropriate UH inhibition prevents over-drive when function is preserved, whereas state-appropriate UH disinhibition enables compensatory engagement when function is impaired. By parameterizing these adjustments with the aperiodic exponent, our study extends the model with a tractable electrophysiological E/I biomarker and provides electrode-specific targets (fronto-parietal region) for individualized neuromodulation.

The aperiodic exponent emerged as a critical spectral feature in our analysis. While traditionally viewed as background noise, this metric is fundamentally linked to the integration of underlying synaptic currents,^33^ contributing to the characteristic 1/*f*-like power spectral density of neural signals.^22^ Physiologically, it serves as a putative marker of the cortical E/I ratio. Recent investigations in epilepsy provide compelling support for this interpretation. Duma *et al*. demonstrated that aperiodic-derived E/I balance correlates with cognitive function in temporal lobe epilepsy,^34^ and tracks the dynamic functional architecture preceding seizure onset.^35^ Kopf *et al*. reported that aperiodic activity indexes neural hyperexcitability in generalized epilepsy.^36^ Furthermore, brain-wide perturbations in E/I balance have been associated with microcircuit organization.^37^ In addition, the E/I ratio is also known to shift following neuromodulation and correlates with treatment efficacy.^38-40^ However, while its utility in characterizing pathology and treatment response is becoming clear, its involvement in post-operative homeostatic regulation—specifically within the UH—has remained unexplored. Our results address this gap. First, we confirmed that the AH is characterized by pathological over-inhibition relative to the UH (manifested as consistently higher aperiodic exponents). Second, and more critically, we demonstrated that the post-operative regulation of the aperiodic exponent in the UH is state-dependent and closely related to motor outcomes.

Preoperative asymmetry between the AH and UH, resulting from hemispheric lesions, appears to impact prognosis. Our findings regarding lateralization are consistent with prior reports of imaging asymmetry.^4^ Such brain asymmetries are well-recognized across various neurological disorders and hold potential as diagnostic biomarkers.^41^ Furthermore, elucidating the organization of asymmetric brain function can inform the development of targeted, lateralized neuromodulatory interventions.^42^ Beyond motor function, lateralized compensation is also evident in other cognitive domains.^43^ While complete functional compensation may not always be attainable, even partial recovery can substantially improve patient quality of life.

Our findings suggest a potential framework for rehabilitation following hemispheric surgery. The model implies that when baseline motor function is preserved, strategies might prioritize stabilization; when impaired, strategies might prioritize facilitation. The aperiodic exponent from routine scalp EEG provides a low-cost biomarker that could potentially assist in stratifying patients and monitoring cortical states. This status-matched, EEG-guided approach provides a hypothesis for individualized rehabilitation and a framework for trials that could randomize polarity by status, using the exponent as a process endpoint.

### Limitation

This study has several limitations. First, our EEG analyses were restricted to NREM sleep periods and to a limited set of spectral features. Other oscillatory features and sleep stages may also shape the post-operative reorganization, but were not systematically examined. It is important to note that the aperiodic exponent is influenced by vigilance states and sleep architecture. We focused on NREM Stage II sleep to ensure signal stationarity. While spectral slope fluctuates with arousal, our intra-subject design—comparing the affected versus UH within the same sleep epoch—mitigates this confound, as global arousal shifts would presumably affect both hemispheres simultaneously. Second, the use of scalp EEG with the standard 10–20 system constrains spatial resolution. As scalp EEG reflects summated extracellular field potentials rather than localized neuronal activity, our results should be interpreted as macroscale cortical states rather than precise laminar or regional mechanisms. In this specific surgical cohort, the operation may alter volume conduction and locally distort signal amplitude and topography. Third, regarding medication effects, while we controlled for the presence of ASMs, we did not perform a detailed quantification of ASM load or serum levels. Therefore, we cannot fully disentangle the specific contribution of pharmacological agents from intrinsic neurophysiological recovery processes. Finally, our design is observational; we infer mechanisms from associations rather than causal perturbation. Despite these constraints, the study identifies robust EEG markers of early functional adaptation and motivates prospective validation.

## Conclusion

In summary, our results are consistent with a model where L-HSP within the UH contributes to motor recovery after hemispheric surgery in a state-dependent manner. The findings suggest that patients with preserved baseline function may benefit from UH stabilization, whereas those with impaired function may benefit from UH facilitation. The aperiodic exponent provides a practical, mechanistically relevant biomarker to track these spectral adjustments. These findings advance a precision-neurorehabilitation framework for catastrophic epilepsy, supporting individualized prognostication and potentially guiding future EEG-informed therapies.

## Supplementary Material

fcag186_Supplementary_Data

## Data Availability

The data that support the findings of this study are available from the corresponding author upon reasonable request. The custom codes generated for this study are provided in the Supplementary material.
