# Supplementary material for: Lateralized excitation–inhibition rebalance correlates with motor recovery following hemispheric surgery
Source: Brain Commun. 2026 May 23;8(3):fcag186. doi: 10.1093/braincomms/fcag186 (PMC13247849; doi:10.1093/braincomms/fcag186)
Supplement: fcag186_Supplementary_Data [file fcag186_supplementary_data.zip › Supplementary Material.pdf]

Supplementary Figure 1. Screening Flow

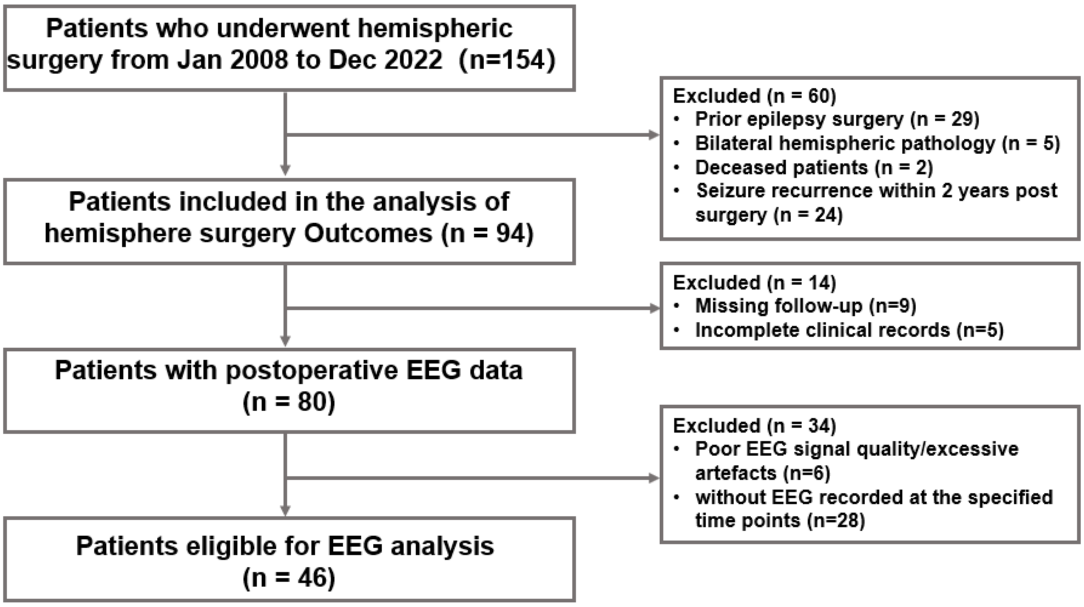

A database of 154 patients who underwent hemispheric surgery between January 2008 and December 2022 was screened. In the initial exclusion step, 60 patients were removed due to a history of prior epilepsy surgery (n = 29), bilateral hemispheric pathology (n = 5), deceased patients (n = 2), or seizure recurrence within 2 years post-surgery (n = 24), resulting in 94 patients eligible for preliminary inclusion. Subsequently, 14 patients were further excluded due to missing follow-up (n = 9) or incomplete clinical records (n = 5), leaving 80 patients with postoperative Electroencephalography (EEG) data. Finally, 34 patients were excluded because of poor EEG signal quality or excessive artefacts (n = 6) or absence of EEG recording at the specified time points (n = 28). This yielded a final cohort of 46 patients eligible for EEG analysis.

Supplementary Figure 2. Validation of the "fixed" aperiodic model choice.

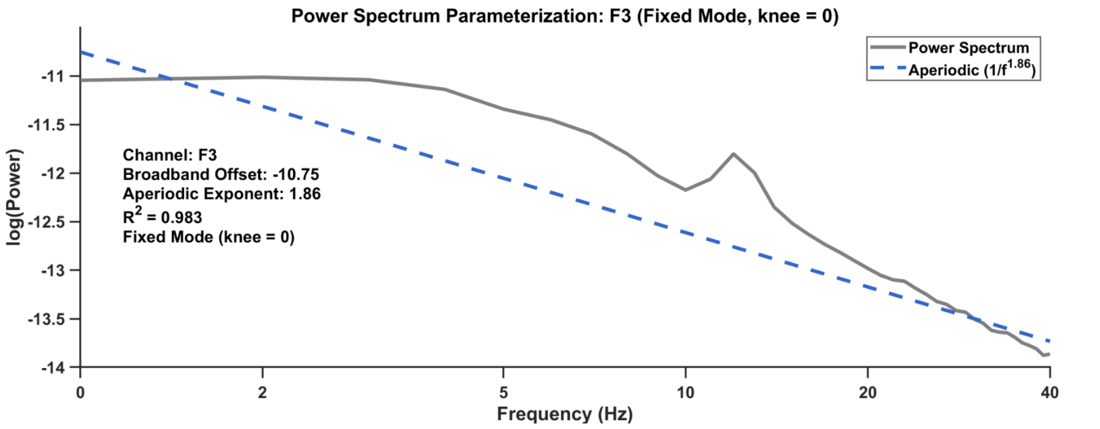

The plot displays the power spectral density (PSD) from a representative electrode (F3) in the preoperative unaffected hemisphere, plotted in log-log space. The signal is derived from a single representative patient and a single electrode. The grey solid line represents the empirical power spectrum, while the blue dashed line indicates the fitted aperiodic component using the "fixed" mode (knee = 0). The model demonstrates a robust linear decay across the analyzed frequency range (1–40 Hz) with excellent goodness of fit (R^2 = 0.983). Peaks extending above the aperiodic line represent distinct oscillatory components. The absence of a characteristic "bend" or plateau in the low-frequency range justifies the exclusion of a knee parameter in this dataset.

Supplementary Figure 3. Preoperative Excitation-Inhibition (E/I) asymmetry

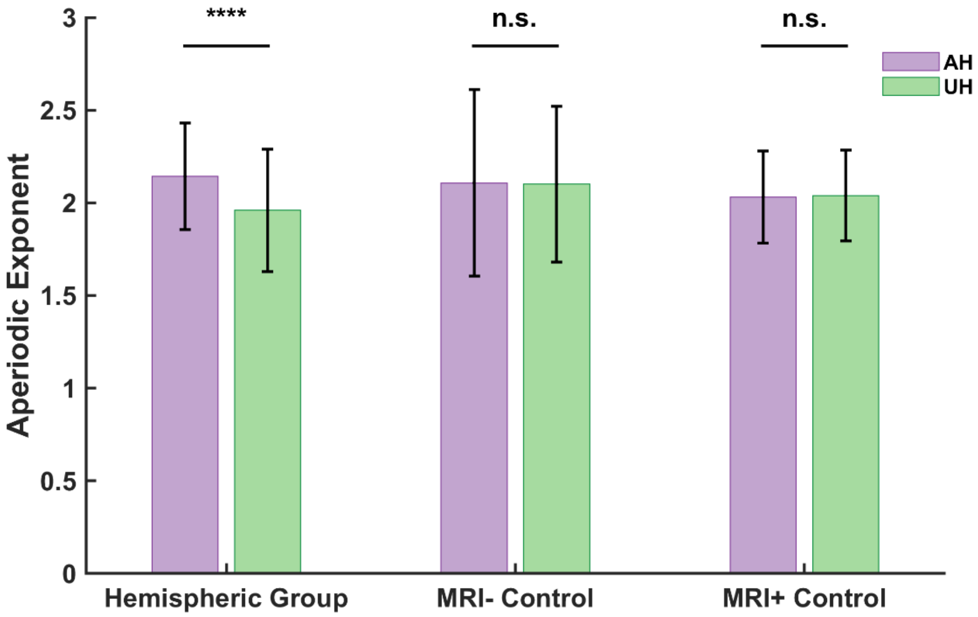

Left. Comparison of preoperative aperiodic exponents between the affected hemisphere (AH, purple) and unaffected hemisphere (UH, green) in the Hemispheric Group (N = 46). The AH showed significantly higher exponents (more inhibition) compared to the UH (Paired t-test, $t(45) = 5.25$, $P < 0.0001$). Middle. Comparison of AH and UH exponents in the aggregate Control Group (N = 23). No significant asymmetry was observed (Paired t-test, $t(22) = 0.10$, $P = 0.9785$). Right. Stratified analysis of the Control Group to rule out lesion burden as a confounder. Controls were divided into Magnetic Resonance Imaging (MRI)-negative (N = 9) and MRI-positive (N = 14) subgroups. Neither the MRI-negative subgroup (Paired t-test, $t(8) = 0.20$, $P = 0.8458$) nor the MRI-positive subgroup (Paired t-test, $t(13) = 0.48$, $P = 0.6360$) exhibited significant inter-hemispheric asymmetry. Single data points represent individual patients for subgroups with N < 10.

Supplementary Table 1: Etiological Spectrum and Electro-Clinical Characteristics of the Hemispheric Group (N=46)

Characteristics	Total (N=46)	Acquired (N=13)	Developmental (N=4)	Progressive (N=29)
**Etiology**				
Perinatal Stroke	8 (17.4%)	8 (61.5%)	-	-
Other Acquired	5 (10.9%)	5 (38.5%)	-	-
Hemimegalencephaly	4 (8.7%)	-	4 (100.0%)	-
Rasmussen Encephalitis	9 (19.6%)	-	-	9 (31.0%)
Sturge-Weber Syndrome	20 (43.5%)	-	-	20 (69.0%)
**Seizure Semiology**				
Focal Motor Seizures	18 (39.1%)	5 (38.5%)	1 (25.0%)	12 (41.4%)
Focal to Bilateral Tonic-Clonic	15 (32.6%)	4 (30.8%)	2 (50.0%)	9 (31.0%)
Epilepsia Partialis Continua	6 (13.0%)	0 (0.0%)	0 (0.0%)	6 (20.7%)
Epileptic Spasms	4 (8.7%)	3 (23.1%)	1 (25.0%)	0 (0.0%)
Focal Non-motor Seizures	3 (6.5%)	1 (7.7%)	0 (0.0%)	2 (6.9%)
EEG Background Activity				
Ipsilateral Slowing	41 (89.1%)	12 (92.3%)	1 (25.0%)	28 (96.6%)
Generalized Slowing	5 (10.9%)	1 (7.7%)	3 (75.0%)	1 (3.4%)
Interictal Epileptiform Discharges				
Strictly Unilateral Discharges	30 (65.2%)	10 (76.9%)	3 (75.0%)	17 (58.6%)
Secondary Bilateral Synchrony	10 (21.7%)	3 (23.1%)	1 (25.0%)	6 (20.7%)
Not Available	6 (13.0%)	0 (0.0%)	0 (0.0%)	6 (20.7%)
Ictal Onset Pattern				
Regional Onset (⩽2 Lobes)	11 (23.9%)	2 (15.4%)	2 (50.0%)	7 (24.1%)
Multilobar/Hemispheric (≥3 Lobes)	10 (21.7%)	3 (23.1%)	2 (50.0%)	5 (17.2%)
Non-localizable / Obscured	25 (54.3%)	8 (61.5%)	0 (0.0%)	17 (58.6%)

Note: Seizure semiology is classified according to the International League Against Epilepsy (ILAE) 2017 guidelines. All patients had focal-onset epilepsy; generalized seizure types (e.g.,

primary generalized tonic-clonic) were exclusionary criteria for hemispherectomy. The "Progressive" etiology group comprises conditions with ongoing neurological deterioration (Rasmussen encephalitis and Sturge-Weber syndrome), whereas "Acquired" refers to static insults (e.g., perinatal stroke). Ictal onset patterns were deemed "Non-localizable/Obscured" in cases with diffuse abnormalities or excessive muscle artifacts affecting lateralization.
